# Supplementary material for: Gut microbial co-abundance networks show specificity in inflammatory bowel disease and obesity
Source: Nat Commun. 2020 Aug 11;11:4018. doi: 10.1038/s41467-020-17840-y (PMC7419557; doi:10.1038/s41467-020-17840-y)
Supplement: Supplementary file 1 — Supplementary Information [file 41467_2020_17840_MOESM1_ESM.pdf]

Gut Microbial Co-abundance Networks Show Specificity in Inflammatory Bowel Disease and Obesity

Chen et al.

TABLE OF CONTENT

Supplementary Note 1 .... **2**

Supplementary Table 1 .... **4**

Supplementary Table 2 .... **5**

Supplementary Figure 1 .... **6**

Supplementary Figure 2 .... **7**

Supplementary Figure 3 .... **8**

Supplementary Figure 4 .... **9**

Supplementary Figure 5 .... **10**

Supplementary Figure 6 .... **11**

Supplementary Figure 7 .... **12**

Supplementary Figure 8 .... **13**

Supplementary Figure 9 .... **14**

Supplementary Figure 10 .... **15**

Supplementary Figure 11 .... **16**

Supplementary Figure 12 .... **17**

Supplementary References **18**

Supplementary Note 1

Gut microbial co-occurrence networks

The metagenomic data of the 2,379 participants from the four cohorts were processed using the same pipeline. After filtering out rare species and pathways present in less than 20% of samples in all cohorts, 134 common bacterial species and 343 microbial pathways were used for network inference. We assessed microbial co-occurrence for the presence/absence of species using the Chi-squared test. At microbial species–level, we identified 6,015 species co-occurrence edges and 19,903 pathway co-occurrence edges significant at False Discovery Rate (FDR)<0.05 in at least one cohort (Supplementary Data 2&4).

Differential microbial co-occurrences

We assessed the similarity of the microbial co-occurrence networks by calculating the heterogeneity of the strength and direction of network edges (i.e. odds ratio [1]) between the four cohorts. Of the 6,015 species co-occurrence edges, 42.0% were significantly variable (Cochran-Q test FDR<0.05) (Supplementary Data 2). Of the 19,903 pathway co-occurrence edges, 47.0% were significantly variable (Cochran-Q test FDR<0.05) (Supplementary Data 4). We also observed a consistent effect for 89.1% of the species co-occurrence edges and 89.3% of the pathway co-occurrence edges from the IBD cohort in 77 IBD patients from the integrative Human Microbiome Project (iHMP-IBD) project [2] (Supplementary Data 2&4).

Differential microbial co-occurrences show cohort-specificity and cohort-specific co-occurrences are enriched in IBD

We further characterized to what extent differential microbial co-occurrences exerted context-specific effects related to disease or health characteristics, i.e. whether the effect size of a co-occurrence in one cohort was significantly different from those in the other three cohorts. In co-occurrence networks, we detected cohort-specific effects for 255 species edges (Supplementary Data 15) and 554 pathway edges

(Supplementary Data 16), with FDRs of 7.6% and 10.0%, respectively. Cohort-specific edges further showed significant enrichment only in the IBD cohort compared to the other three cohorts (300OB, LLD and 500FG), including 233 IBD-specific species edges (Fisher's test $P=1.1x10^{-104}$) and 473 IBD-specific pathway edges (Fisher's test $P=5.5x10^{-197}$). We could also replicate 79.4% of IBD-specific species edges and 84.8% of IBD-specific pathway edges in the iHMP-IBD cohort (Cochran-Q test P>0.05, Supplementary Data 2&4).

Key species and pathways in IBD-specific co-occurrences

We checked to what extent cohort-specific co-occurrences were enriched for specific pathways or species, which we consider to be key species or pathways (we require at least 10 cohort-specific connections for key species and 45 for key pathways). Our analysis only detected 5 key species in the IBD co-occurrence network (Supplementary Data 15&16). These species mainly involved in phyla *Firmicutes*, including *Ruminococcaceae bacterium_D16*, *Lachnospiraceae bacterium_3_1_46FAA*, *Ruminococcus lactaris* ect (Supplementary Data 15&16).

Supplementary Table 1: Summary of sub-phenotypes in the IBD and obesity.

	IBD (n = 496)		
**Phenotypes**	**CD (n = 276)**	**UC (n = 189)**	**IBDU (n = 31)**
Age mean (range)	41.2 (18 - 81)	46.6 (19 - 82)	44.2 (19 - 76)
**Disease location**			
Colon n (%)	59 (22)	189 (100)	31 (100)
Ileum n (%)	92 (35)	0 (0)	0 (0)
Both n (%)	112 (43)	0 (0)	0 (0)
**Active disease n (%)**	69 (25)	46 (25)	6 (24)
**Antibiotics yes (%)**	58 (21)	32 (17)	5 (16)
**IBD-medication**			
Mesalazines yes(%)	25 (9)	123 (65)	23 (74)
Steroids yes (%)	46 (17)	31 (16)	4 (13)
Immunosuppresants yes (%)	129 (47)	65 (34)	7 (23)
Anti-TNFalpha yes (%)	101 (37)	19 (10)	3 (10)
Thiopurines yes (%)	97 (35)	52 (28)	4 (13)
Other biologicals yes (%)	3 (1)	0 (0)	0 (0)
**Other medications**			
ACE-inhibitor yes (%)	10 (4)	10 (5)	4 (13)
angII-receptor antagonist yes (%)	4 (1)	5 (3)	1 (3)
Beta-blockers yes (%)	15 (5)	10 (5)	6 (19)
Bisphosphonates yes (%)	6 (2)	5 (3)	0 (0)
Iron supplementation yes (%)	7 (3)	6 (3)	0 (0)
Folic acid yes (%)	26 (9)	1 (1)	2 (6)
Laxatives yes (%)	20 (7)	6 (3)	3 (10)
Metformin yes (%)	2 (1)	4 (2)	1 (3)
NSAID yes (%)	13 (5)	4 (2)	4 (13)
Opiat yes (%)	19 (7)	1 (1)	1 (3)
Platelet aggregation inhibitor yes (%)	12 (4)	11 (6)	3 (10)
PPI yes (%)	66 (24)	28 (15)	7 (23)
SSRI-antidepressant yes (%)	5 (2)	2 (1)	2 (6)
Statin yes (%)	9 (3)	14 (7)	3 (10)
Thiazide diuretic yes (%)	6 (2)	9 (5)	1 (3)

	**300OB (n = 298)**
Age mean (range)	67.1 (54 - 80)
Diabetes yes (%)	35 (12)
Atherosclerotic plaque yes (%)	139 (47)

Supplementary Table 2. Microbial pathway associations to fasting plasma metabolites in obesity. 298 samples were included in the analysis and Spearman correlation was conducted to access the correlation between microbial pathway abundance and plasma levels of glucose and insulin with adjustment of age and sex.

Pathway	Plasma metabolites	Coefficient	P value
PWY-2942: L-lysine biosynthesis III	glucose	0.117163851	0.04403832
PWY-5097: L-lysine biosynthesis VI	glucose	0.148923037	0.010354671
PWY-6151: S-adenosyl-L-methionine cycle I	glucose	0.167177501	0.003961159
THRESYN-PWY: superpathway of L-threonine biosynthesis	glucose	0.124700509	0.032031956
HOMOSER-METSYN-PWY: L-methionine biosynthesis I	insulin	-0.128748486	0.026567724
MET-SAM-PWY: superpathway of S-adenosyl-L-methionine biosynthesis	insulin	-0.142087939	0.014313333
METSYN-PWY: L-homoserine and L-methionine biosynthesis	insulin	-0.137604047	0.017719186
PWY-3001: superpathway of L-isoleucine biosynthesis I	insulin	-0.116186809	0.045474291
PWY-5347: superpathway of L-methionine biosynthesis (transsulfuration)	insulin	-0.145478914	0.012133817
THRESYN-PWY: superpathway of L-threonine biosynthesis	insulin	-0.132846239	0.022086831

Supplementary Figure 1. Graphical summary of the present study.

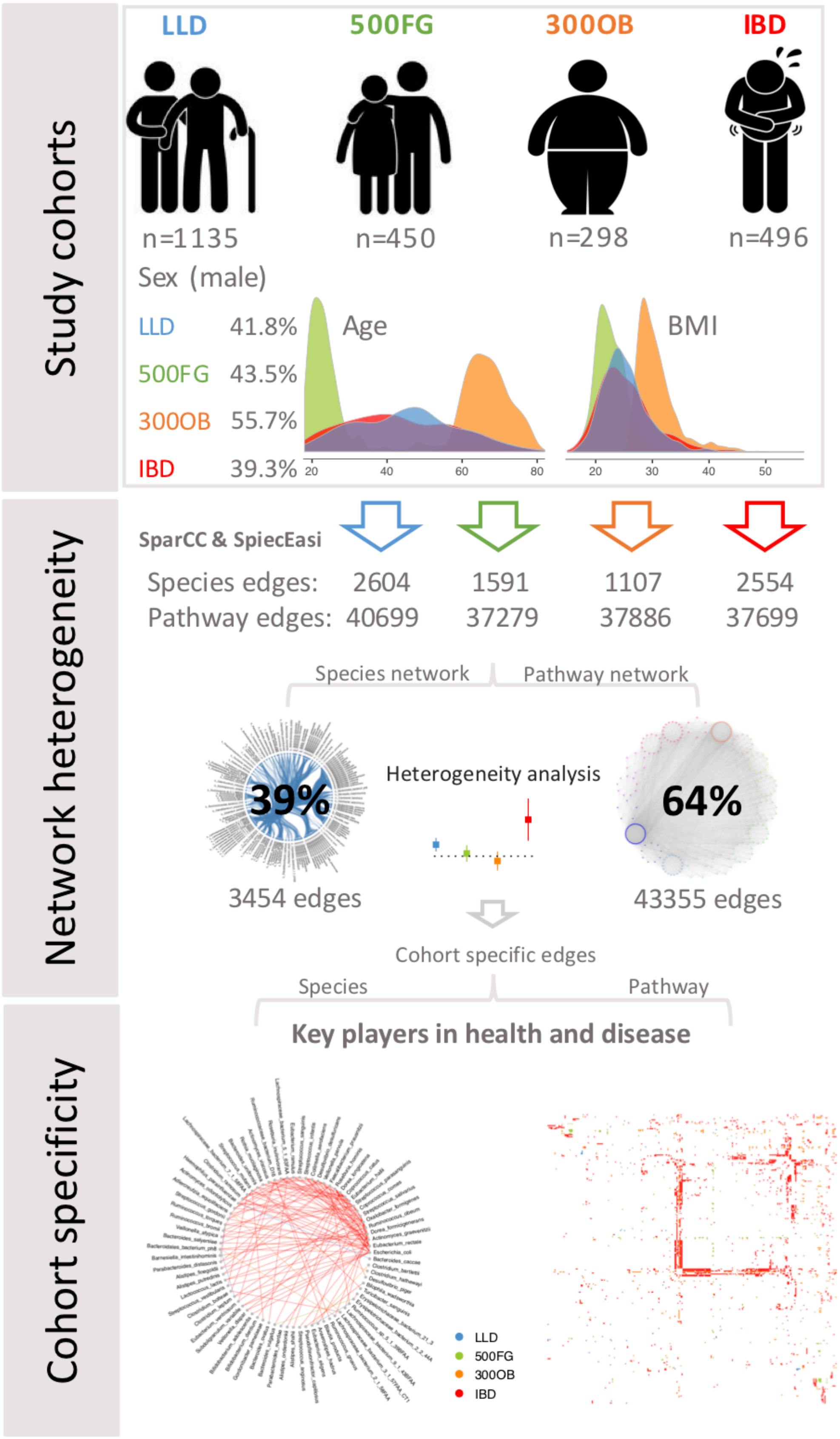

Supplementary Figure 2. Principal component analysis of microbial species and pathways. A. PCoA (Bray-Curtis distance matrix) of 134 species that are present in >20% of samples in at least one cohort. B. PCA (Euclidean distance matrix) of 343 pathway that are present in >20% of samples in at least one cohort. N=2379 independent samples were involved (N_{LLD}=1135, N_{500FG}=450, N_{300OB}=298, N_{IBD}=496). The one-sided ANOVA test was applied to assess microbial compositional difference between cohorts. Dots represent the value per each sample in different PCs. Boxplot shows the median and interquartile range (25th and 75th). Whiskers show the 1.5*IQR range. The upper and lower whiskers extend the largest and smallest value no further than 1.5*IQR, respectively. Outliers are plotted individually (Source data is provided as a Source Data file).

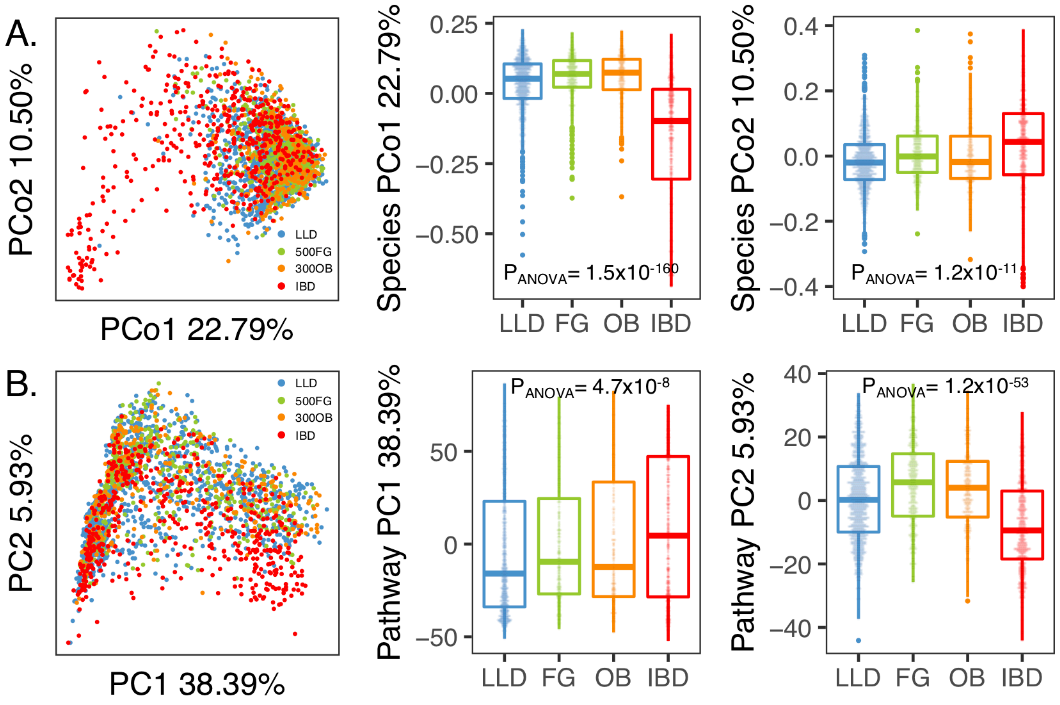

Supplementary Figure 3. Overlapping of microbial co-occurrence and co-abundance networks. 82.1% of species co-abundances and 29.6% of pathways co-abundances also exerted co-occurrences.

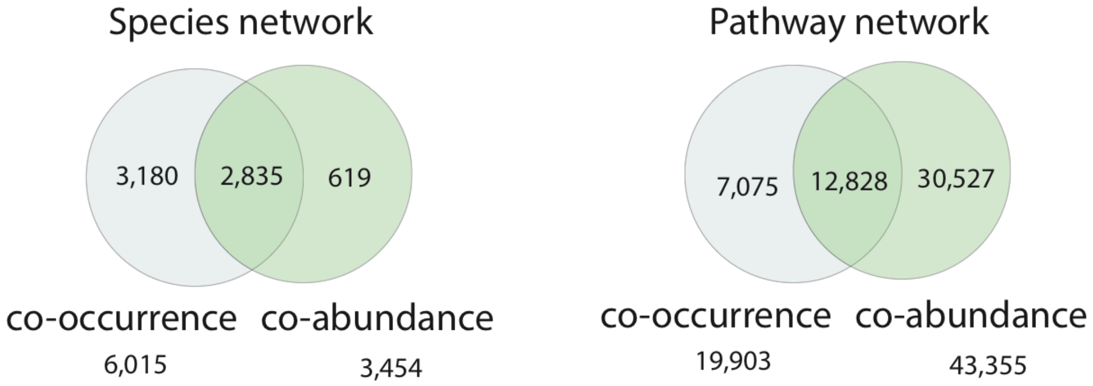

Supplementary Figure 4. 120 cohort-specific species co-abundances. Each dot indicates one species. Each line represents one cohort-specific co-abundance relationship. The colour key for cohort-specific co-abundance is shown. The three key species in IBD are marked in black.

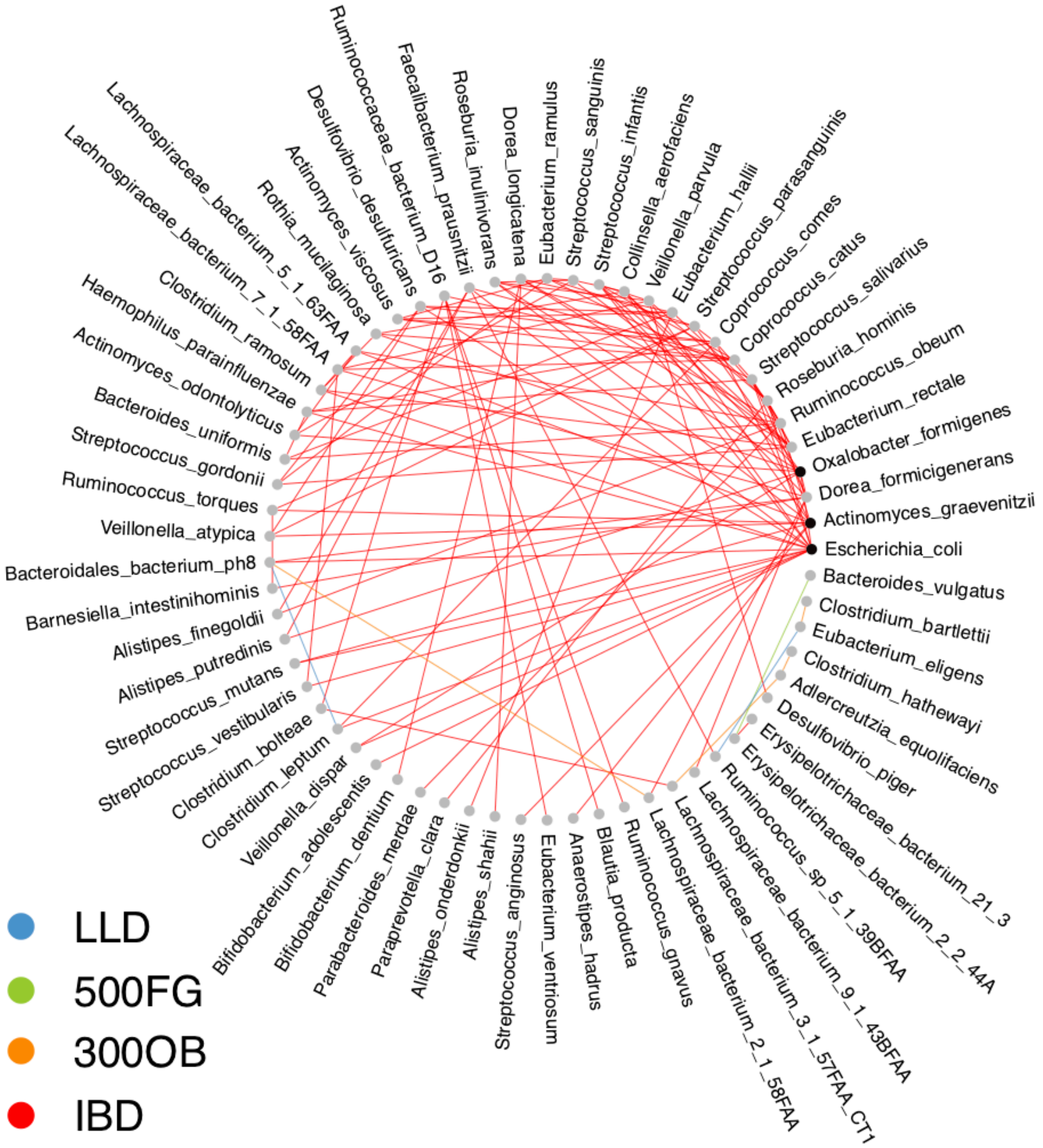

Supplementary Figure 5. 1,448 cohort-specific pathway co-abundances. Both the horizontal and vertical axes are pathways coloured by metabolic category. Each cell represents a cohort-specific co-abundance relationship, and cell colours vary with cohort. The colours indicating cohort-specificity and metabolic categories are shown in the legends. Hierarchical clustering analysis reveals enrichment for cohort-specificity. The one key pathway in obesity and the four key pathways in IBD are marked in orange and red, respectively.

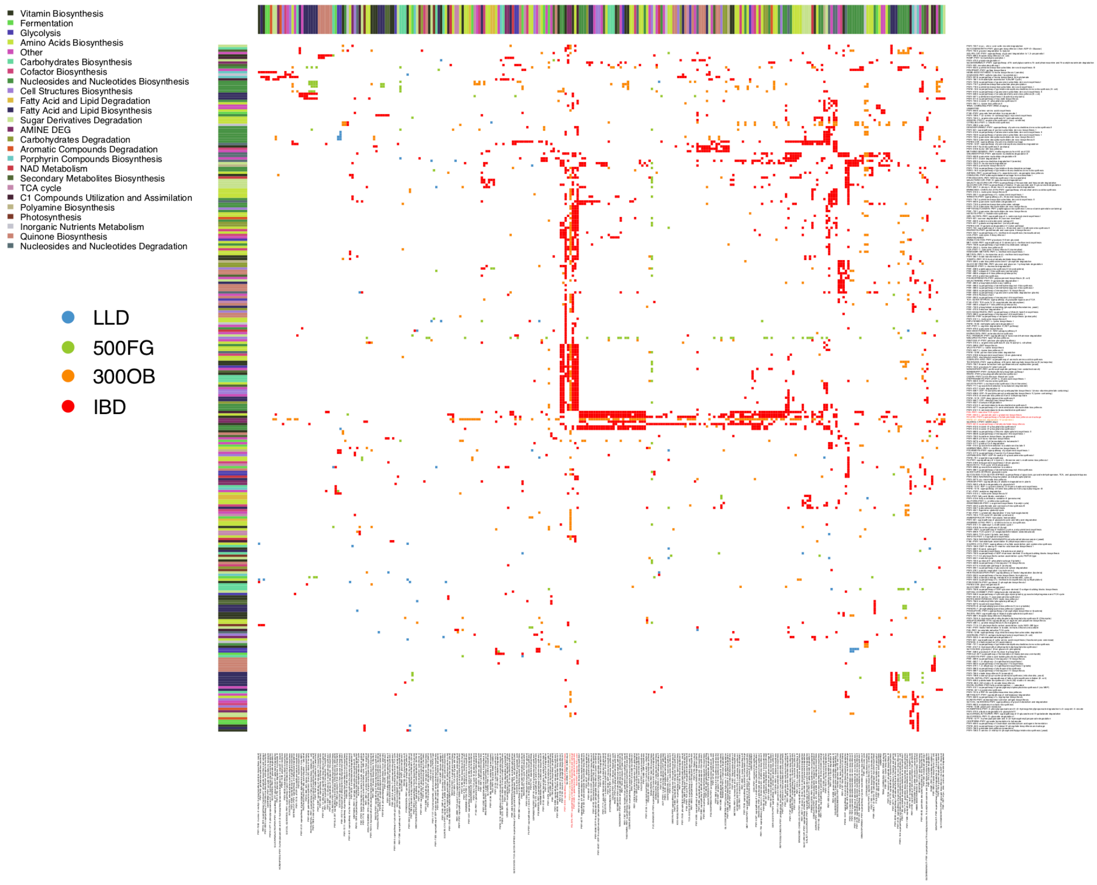

Supplementary Figure 6. Replication of the IBD network using longitudinal data from the iHMP-IBD cohort. We assessed the replication rate of IBD co-abundances in the iHMP-IBD cohort and their stability between first and last time points of data collection. Each dot represents one co-abundance. Upper panel shows species co-abundances. Lower panel show pathway co-abundances. Both the X- and Y-axes represent correlation coefficients of co-abundances. Red dots indicate microbial co-abundances that show a difference in effect size between the first and last time points at P<0.05 by using Cochran's Q test.

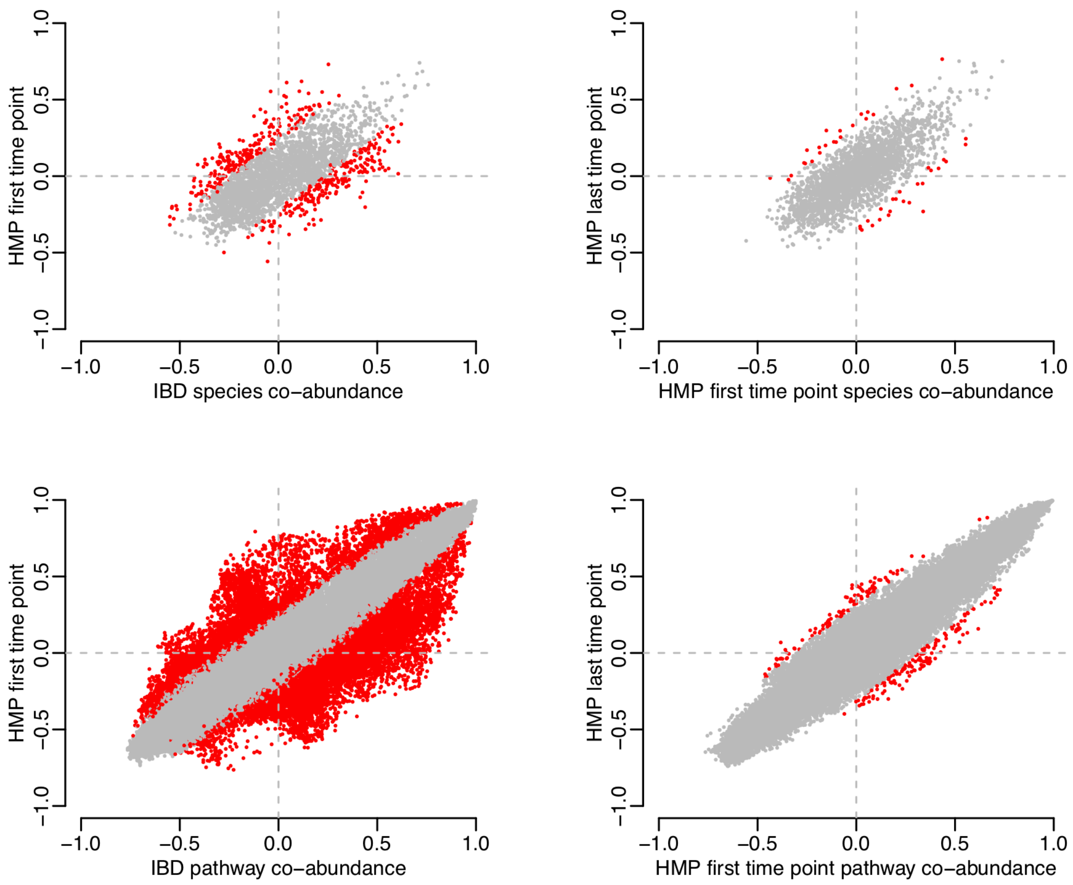

Supplementary Figure 7. IBD co-abundances in relation to sub-phenotypes. We assessed whether microbial co-abundances in IBD showed a difference between IBD subtypes (UC vs. CD), disease activities (inflammation vs. no inflammation) and locations (ileum vs. colon) and with the usage of PPIs and antibiotics. Upper panel shows species co-abundances. Lower panel shows pathway co-abundances. Each dot represents one co-abundance. Both the X- and Y-axes represent correlation coefficients of co-abundances. Red dots indicate microbial co-abundances that show a difference in effect size between sub-phenotypes at FDR<0.05 by using Cochran's Q test.

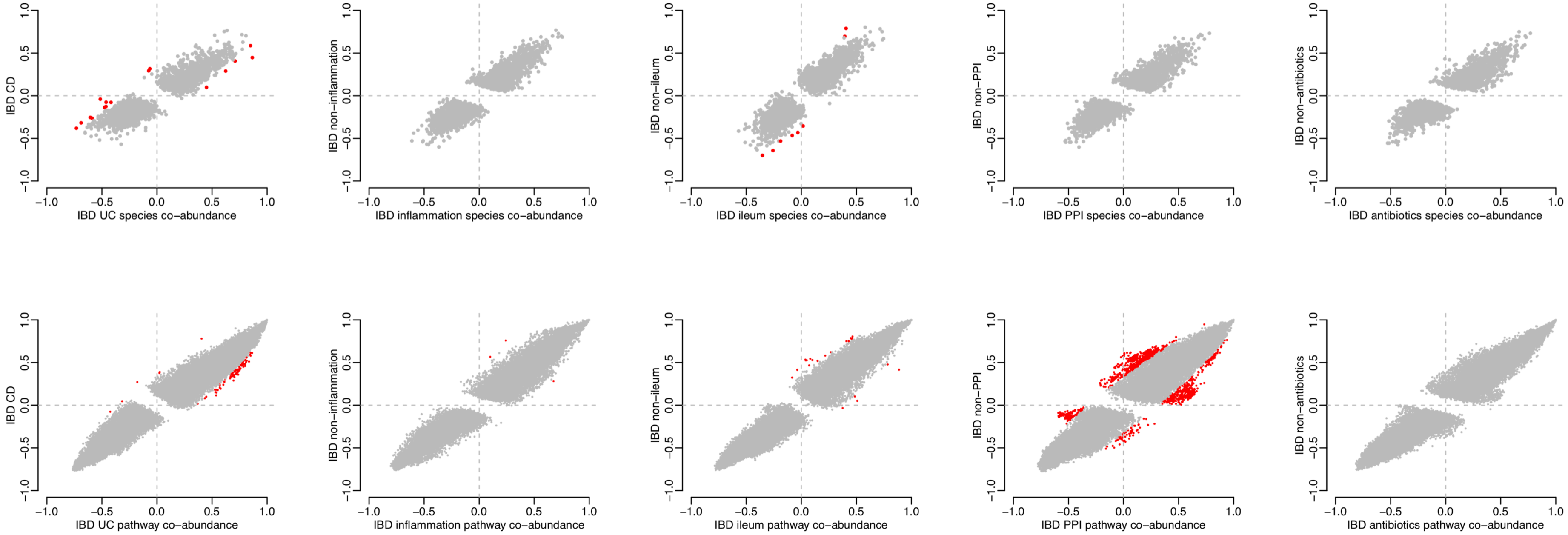

Supplementary Figure 8. Replication of the obesity network in 134 obese individuals from the LLD cohort. We compared co-abundance strengths in terms of correlation coefficients in the 300OB cohort with values for 134 obese individuals from the LLD cohort with similar ages and BMIs. The X-axis represents the estimated correlation coefficients in the 300OB cohort. The Y-axis represents the estimated correlation coefficients in obese individuals from the LLD cohort. Upper panel shows species co-abundances. Lower panel shows pathway co-abundances. Each dot is one co-abundance. Red dots indicate microbial co-abundances that show a difference in their effect size between the discovery and replication at P<0.05 by using Cochran's Q test.

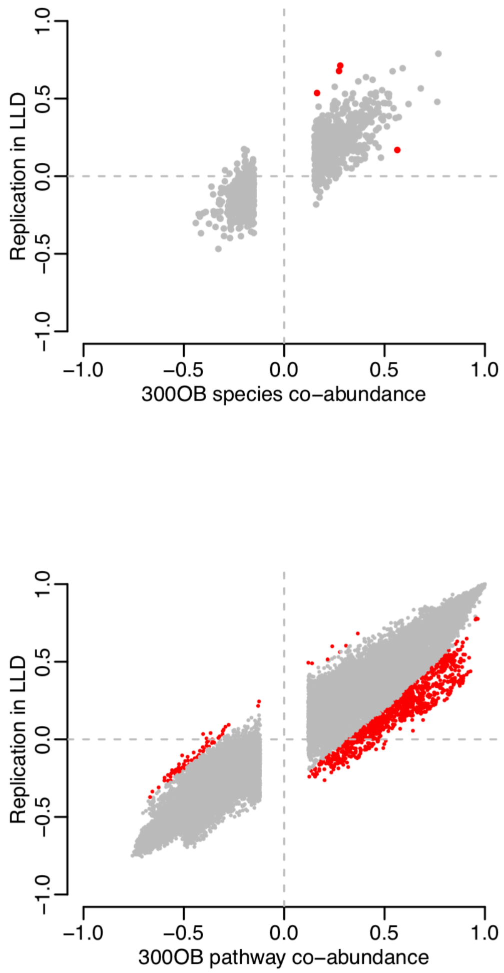

Supplementary Figure 9. Obesity co-abundances in relation to phenotypes. We further assessed whether microbial co-abundances in 300OB showed difference between patients with and without diabetes and atherosclerotic plaque. Upper panel shows species co-abundances. Lower panel shows pathway co-abundances. Each dot represents one co-abundance. Both the X- and Y-axes represent correlation coefficients of co-abundances. Red dots represent microbial co-abundances that show a difference in their effect size between subtypes at FDR<0.05 by using Cochran's Q test.

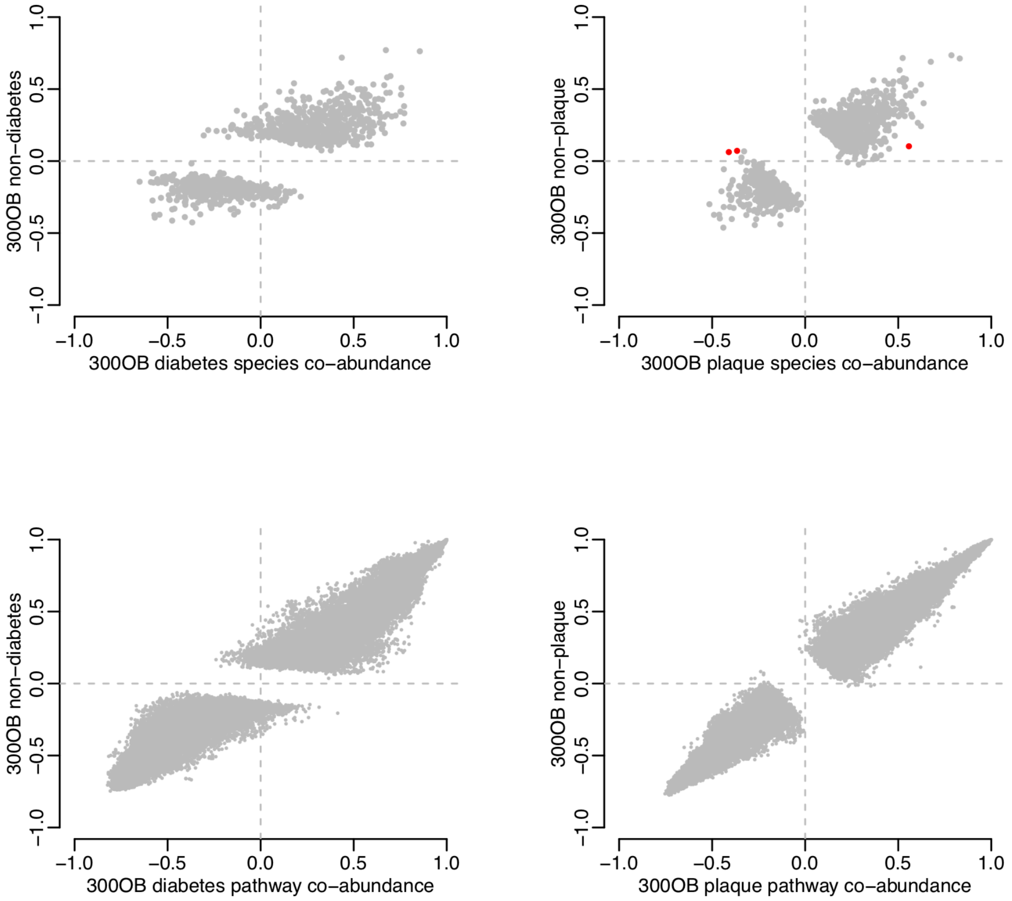

Supplementary Figure 10. Assessing cohort specific co-abundances. An interquartile range–based method was used to detect cohort-specific co-abundances.

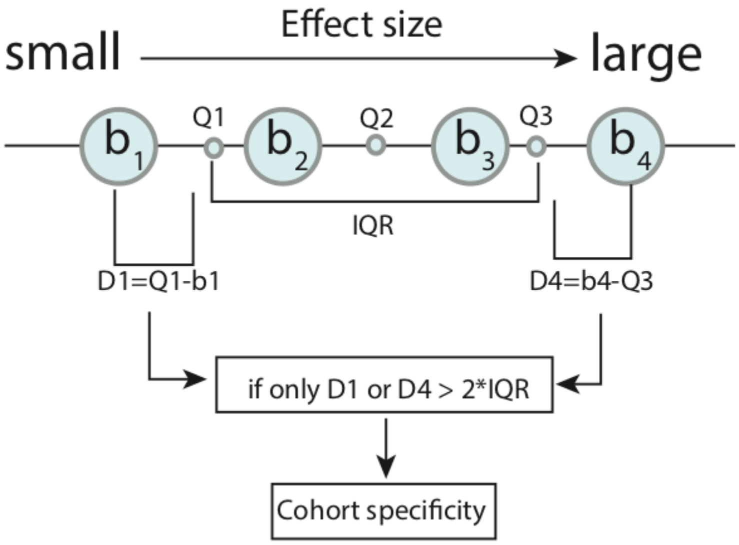

Supplementary Figure 11. Assessing the impact of age and sex on co-abundances. We used partial correlation to regress out the impact of age and sex on co-abundances.

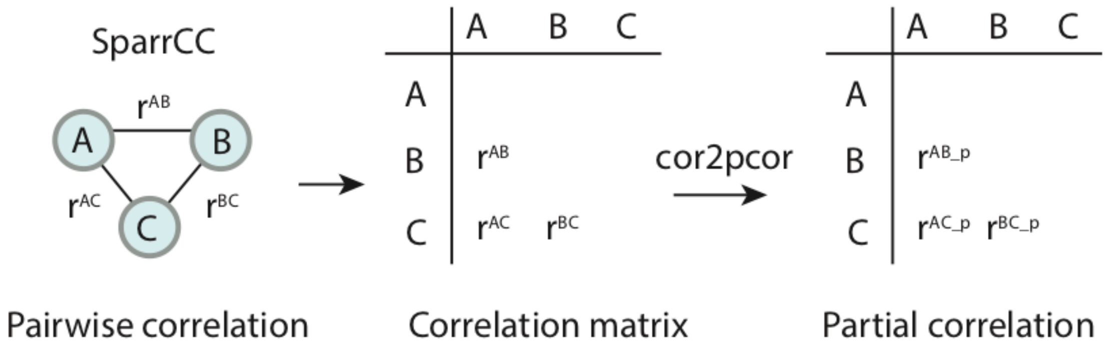

Supplementary Figure 12. Age and sex have limited impact on the effect size and direction of co-abundances. The figure shows the comparison of the correlation coefficient of each co-abundance before and after regressing out age and sex using partial correlation. Upper panel shows species co-abundances. Lower panel shows pathway co-abundances. Each dot represents one co-abundance. Both the X- and Y-axis represent correlation coefficients of co-abundances.

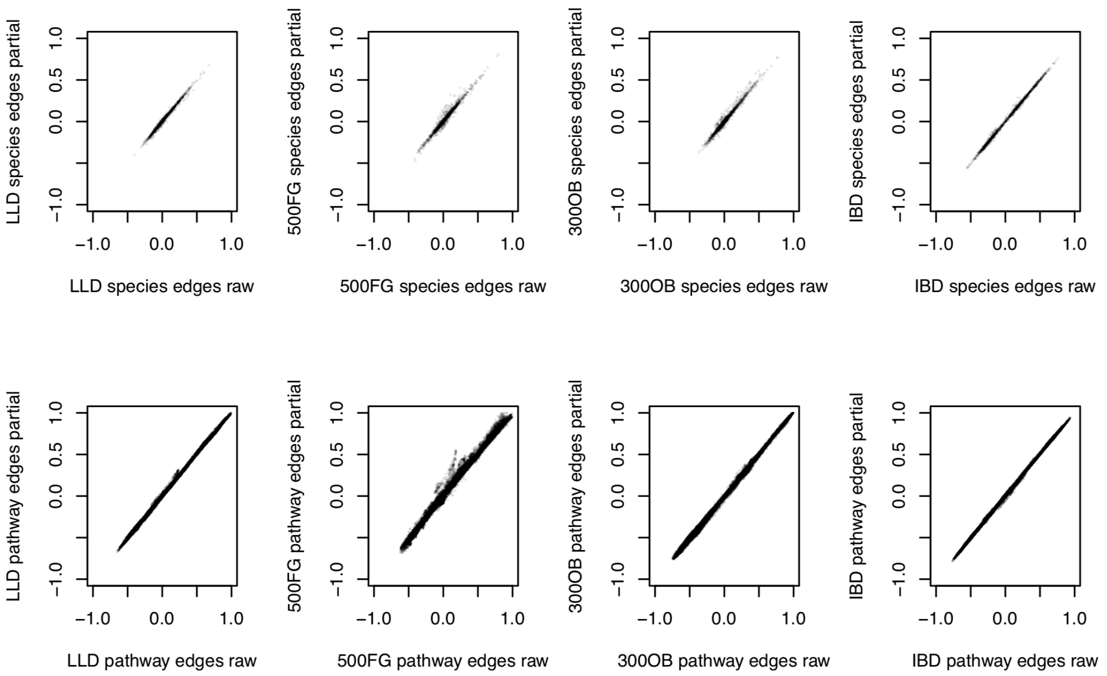

Supplementary References

1. Valenzuela C: [2 solutions for estimating odds ratios with zeros]. Rev Med Chil 1993, 121:1441-1444.
2. Proctor LM, Network IHiR: The Integrative Human Microbiome Project: Dynamic Analysis of Microbiome-Host Omics Profiles during Periods of Human Health and Disease. Cell Host & Microbe 2014, 16:276-289.
