# Supplementary material for: Gut microbial co-abundance networks show specificity in inflammatory bowel disease and obesity
Source: Nat Commun. 2020 Aug 11;11:4018. doi: 10.1038/s41467-020-17840-y (PMC7419557; doi:10.1038/s41467-020-17840-y)
Supplement: Supplementary file 3 — Description of Additional Supplementary Files [file 41467_2020_17840_MOESM3_ESM.pdf]

Description of Additional Supplementary Files

File name: Supplementary Data 1
Description: Species co-abundance network. SparCC was applied to assess the correlation coefficient of microbial co-abundances and Cochran-Q was conducted to assess the heterogeneity of correlation coefficients.

File name: Supplementary Data 2
Description: Species co-occurrence network. Co-occurrence strength was indicated by odds-ratio and Cochran-Q was conducted to assess the heterogeneity of odds-ratios.

File name: Supplementary Data 3
Description: Pathway co-abundance network. SparCC was applied to assess the correlation coefficient of microbial co-abundances and Cochran-Q was conducted to assess the heterogeneity of correlation coefficients.

File name: Supplementary Data 4
Description: Pathway co-occurrence network. Co-occurrence strength was indicated by odds-ratio and Cochran-Q was conducted to assess the heterogeneity of odds-ratios.

File name: Supplementary Data 5
Description: Summary of species co-abundance network. Differential microbial species abundance among cohorts was assessed with ANOVA test with correction for age and sex.

File name: Supplementary Data 6
Description: Summary of pathway co-abundance network. Differential microbial pathway abundance among cohorts was assessed with ANOVA test with correction for age and sex.

File name: Supplementary Data 7
Description: Replication of IBD species co-abundance. SparCC was applied to access the correlation coefficient of microbial co-abundances and Cochran-Q was conducted to access the heterogeneity of correlation coefficients.

File name: Supplementary Data 8
Description: Replication of IBD pathway co-abundance. SparCC was applied to assess the correlation coefficient of microbial co-abundances and Cochran-Q was conducted to assess the heterogeneity of correlation coefficients.

File name: Supplementary Data 9
Description: Relevance of phenotypes to IBD species co-abundance. SparCC was applied to assess the correlation coefficient of microbial co-abundances and Cochran-Q was conducted to assess the heterogeneity of correlation coefficients.

File name: Supplementary Data 10
Description: Relevance of phenotypes to IBD pathway co-abundance. SparCC was applied to assess the correlation coefficient of microbial co-abundances and Cochran-Q was conducted to assess the heterogeneity of correlation coefficients.

File name: Supplementary Data 11
Description: Pathway contributed by dominant species in each cohort.

File name: Supplementary Data 12
Description: Correlation between species and pathway abundance. Spearman correlation was applied to assess correlation between microbial species and pathway abundance with adjustment of age and sex.

File name: Supplementary Data 13
Description: Replication and relevance of phenotypes to 300OB species co-abundance. SparCC was applied to assess the correlation coefficient of microbial co-abundances and Cochran-Q was conducted to assess the heterogeneity of correlation coefficients.

File name: Supplementary Data 14
Description: Replication and relevance of phenotypes to 300OB pathway co-abundance. SparCC was applied to assess the correlation coefficient of microbial co-abundances and Cochran-Q was conducted to assess the heterogeneity of correlation coefficients.

File name: Supplementary Data 15
Description: Summary of species co-occurrence network.

File name: Supplementary Data 16
Description: Summary of pathway co-occurrence network.
