# Supplementary material for: Gut microbial co-abundance networks show specificity in inflammatory bowel disease and obesity
Source: Nat Commun. 2020 Aug 11;11:4018. doi: 10.1038/s41467-020-17840-y (PMC7419557; doi:10.1038/s41467-020-17840-y)
Supplement: Supplementary file 20 — Reporting Summary [file 41467_2020_17840_MOESM20_ESM.pdf]

natureresearch

Corresponding author(s): Jingyuan Fu
Last updated by author(s): Jun 25, 2020

Reporting Summary

Nature Research wishes to improve the reproducibility of the work that we publish. This form provides structure for consistency and transparency in reporting. For further information on Nature Research policies, see Authors & Referees and the Editorial Policy Checklist .

Statistics

For all statistical analyses, confirm that the following items are present in the figure legend, table legend, main text, or Methods section.

n/a	Confirmed	
☐	☒	The exact sample size (*n*) for each experimental group/condition, given as a discrete number and unit of measurement
☐	☒	A statement on whether measurements were taken from distinct samples or whether the same sample was measured repeatedly
☐	☒	The statistical test(s) used AND whether they are one- or two-sided<br>*Only common tests should be described solely by name; describe more complex techniques in the Methods section.*
☐	☒	A description of all covariates tested
☐	☒	A description of any assumptions or corrections, such as tests of normality and adjustment for multiple comparisons
☐	☒	A full description of the statistical parameters including central tendency (e.g. means) or other basic estimates (e.g. regression coefficient) AND variation (e.g. standard deviation) or associated estimates of uncertainty (e.g. confidence intervals)
☐	☒	For null hypothesis testing, the test statistic (e.g. *F*, *t*, *r*) with confidence intervals, effect sizes, degrees of freedom and *P* value noted<br>*Give P values as exact values whenever suitable.*
☒	☐	For Bayesian analysis, information on the choice of priors and Markov chain Monte Carlo settings
☒	☐	For hierarchical and complex designs, identification of the appropriate level for tests and full reporting of outcomes
☐	☒	Estimates of effect sizes (e.g. Cohen's *d*, Pearson's *r*), indicating how they were calculated

Our web collection on statistics for biologists contains articles on many of the points above.

Software and code

Policy information about availability of computer code

Data collection	No software was used for data collection.
Data analysis	All data analyses were conducted using publicly available tools. For this study the following software was used: kneadData (v0.4.6.1), Bowtie2 (v2.1.0), MetaPhlAn2 (v2.7.2), HUMAnN2 (v0.10.0), SparCC Python package, R (v3.5.2), SpiecEasi R package (v1.0.6) and meta R package (v4.9.5). Code used for generating the microbial abundance profiles is publicly available at: [https://github.com/GRONINGEN-MICROBIOME-CENTRE/Groningen-Microbiome/blob/master/Scripts/Metagenomics_pipeline_v1.md]. Code used for the statistical analyses is publicly available at: [https://github.com/GRONINGEN-MICROBIOME-CENTRE/Groningen-Microbiome/tree/master/Projects/Microbial%20co-abundance%20network].

For manuscripts utilizing custom algorithms or software that are central to the research but not yet described in published literature, software must be made available to editors/reviewers. We strongly encourage code deposition in a community repository (e.g. GitHub). See the Nature Research guidelines for submitting code & software for further information.

Data

Policy information about availability of data

All manuscripts must include a data availability statement. This statement should provide the following information, where applicable:

- Accession codes, unique identifiers, or web links for publicly available datasets
- A list of figures that have associated raw data
- A description of any restrictions on data availability

All relevant data supporting the key findings of this study are available within the article and its Supplementary Information files. Data underlying Figure 5C and Supplementary Figure 2 are provided as a Source Data file. Data underlying all the other Figures are provided in Supplementary Data files. The raw metagenomic sequencing data and human phenotypes (i.e. age and sex) used for the analysis presented in this study are available from the European Genome-phenome Archive and National Center for Biotechnology Information data repositories: LifeLines Deep cohort [https://www.ebi.ac.uk/ega/datasets/EGAD00001001991], 1000 IBD cohort [https://www.ebi.ac.uk/ega/datasets/EGAD00001004194], 300OB cohort [https://ega-archive.org/dacs/EGAC00001001143], 500FG cohort [https://

www.ncbi.nlm.nih.gov/bioproject/?term=PRJNA319574]. The iHMP data is available via: [https://ibdmdb.org/tunnel/public/summary.html]. Data access is subject to local rules and regulations.

Field-specific reporting

Please select the one below that is the best fit for your research. If you are not sure, read the appropriate sections before making your selection.

☒ Life sciences ☐ Behavioural & social sciences ☐ Ecological, evolutionary & environmental sciences

For a reference copy of the document with all sections, see nature.com/documents/nr-reporting-summary-flat.pdf

Life sciences study design

All studies must disclose on these points even when the disclosure is negative.

Sample size	All metagenomic samples (n=2,379) from four cohorts: a population-based cohort (LifeLines-Deep, n=1,135), a young adult cohort (500FG, n=450), a clinically diagnosed obesity cohort (300OB, n=298) and an inflammatory bowel disease cohort (IBD, n=496) with phenotypes (age and sex) available were selected. Moreover, an extra IBD cohort with 77 subjects was included as a replication cohort. In order to ensure the analysis power, the study includes as much as subjects as possible. Thus no sample size calculation was performed.
Data exclusions	Only samples with low quality of metagenomics sequencing were excluded.
Replication	For microbial networks in IBD, we used 77 IBD samples available from the iHMP cohort as replication. The replication rate was 90.6% for species co-abundances and 99.6% for pathway co-abundances. For the network in obesity, we used 134 LifeLines-Deep samples with matched age and BMI as replication. The replication rate is 89.5% for species co-abundances and 87.0% for pathway co-abundances.
Randomization	This is human cohort-based analysis. The sample collection and sequencing were performed in a random order. No extra randomization was done for this study.
Blinding	This study is a human cohort based, observational study. Thus no blinding was performed.

Reporting for specific materials, systems and methods

We require information from authors about some types of materials, experimental systems and methods used in many studies. Here, indicate whether each material, system or method listed is relevant to your study. If you are not sure if a list item applies to your research, read the appropriate section before selecting a response.

Materials & experimental systems

n/a	Involved in the study	
☒	☐	Antibodies
☒	☐	Eukaryotic cell lines
☒	☐	Palaeontology
☒	☐	Animals and other organisms
☐	☒	Human research participants
☒	☐	Clinical data

Methods

n/a	Involved in the study	
☒	☐	ChIP-seq
☒	☐	Flow cytometry
☒	☐	MRI-based neuroimaging

Human research participants

Policy information about studies involving human research participants

Population characteristics	The study has included four cohorts from the Netherlands: a population-based cohort LifeLines-Deep, n=1,135, 58.20% female, the mean age (SD) of participants is 45.04 (13.60) years and their mean BMI is 25.26 (4.18); a young adult cohort 500FG, n=450, 56.50% female, the mean age of participants is 27.43 (12.35) years and their mean BMI is 22.70 (2.72); a clinically diagnosed obesity cohort 300OB, n=298, 44.30% female, the mean age of participants is 67.07 (5.39) years and their mean BMI is 30.73 (3.48); and an inflammatory bowel disease cohort IBD, n=496, 60.70% female, the mean age of participants is 43.45 (14.52) years and their mean BMI is 25.55 (5.17).
Recruitment	The LifeLines-DEEP cohort is a random subset of the population-based Lifelines cohort. The 500FG cohort is also population-based cohort without any specific selection. For the 300OB cohort, 302 individuals aged >55 years and BMI >27 kg/m2 were enrolled at the Radboud University Medical Center, Nijmegen, the Netherlands. The IBD cohort consists of patients with IBD recruited at the specialized IBD outpatient clinic of the University Medical Center Groningen in the Netherlands. IBD diagnosis was made based on accepted radiological, endoscopic and histopathological evaluation. Moreover, all participants were collected in the Netherlands. Thus the reported results could be biased towards disease sub-phenotypes and region-specific microbiome. However, independent replication carried in both the iHMP and LLD sub cohort illustrated the robustness of results.
Ethics oversight	All participants signed an informed consent form prior to sample collection. Institutional ethics review board (IRB) approval was available for all four cohorts: the Lifelines DEEP (ref. M12.113965) and the IBD (IRB-number 2008.338) cohorts were approved by the UMCG IRB and the 500FG study (NL42561.091.12, 2012/550) and 300OB (NL46846.091.13) cohorts were approved by the Ethical Committee of Radboud University Nijmegen.

Note that full information on the approval of the study protocol must also be provided in the manuscript.
